# Baseline assessment of front-line health system capacity in vector-borne disease surveillance and response in Papua New Guinea

**DOI:** 10.1371/journal.pgph.0004108

**Published:** 2025-04-30

**Authors:** Rachael J. Farquhar, Zebedee Kerry, Yasmin Mohamed, Christopher Morgan, Annie Dori, Samuel McEwen, Diana Timbi, Willie Porau, Nakapi Tefuarani, William Pomat, Leo Makita, Moses Laman, Leanne J. Robinson

**Affiliations:** 1 Burnet Institute, Melbourne, Australia; 2 Papua New Guinea Institute of Medical Research, Port Moresby, Papua New Guinea; 3 Murdoch Children’s Research Institute, Melbourne, Australia; 4 Department of Paediatrics, University of Melbourne, Melbourne, Australia; 5 Jhpiego, the Johns Hopkins University affiliate, Washington, United States of America; 6 National Malaria Control Program, National Department of Health, Port Moresby, Papua New Guinea,; 7 National Department of Health, Central Public Health Laboratory, Port Moresby, Papua New Guinea; 8 School of Medicine and Health Sciences, University of Papua New Guinea, Port Moresby, Papua New Guinea; 9 Walter and Eliza Hall Institute, Melbourne, Australia; Menzies School of Health Research, AUSTRALIA

## Abstract

International public health surveillance and timely response cannot exist without strengthened local surveillance and response systems. Supporting front-line healthcare workers to embed the use of innovative electronic health information systems into adaptable and sustainable processes within their contexts is essential for response to ongoing vector-borne diseases (VBDs) and emerging infectious diseases in resource-constrained settings such as Papua New Guinea (PNG). Baseline health service assessments were conducted at eight provinces to inform the implementation of the STRIVE-Tupaia platform. The STRIVE-Tupaia platform is an innovative electronic real-time data aggregation platform in PNG where healthcare workers are able to review, interpret and respond to febrile illness, molecular diagnostic and vector surveillance data to support evidence-based decision making. Baseline health service assessments involved semi-structured interviews and structured observations of facility procedures. Quantitative data were analyzed using Microsoft Excel, while the qualitative data were thematically coded in NVivo12 (QSR International Pty Ltd). A deductive analysis focussed on the health systems barriers and enablers using the WHO’s seven component health systems framework. An inductive analysis explored these impacts for vector-borne disease services specifically. Results indicated barriers to VBD reporting, notification and response including limited training, infrastructure challenges, overstretched workforce and limited governance support. Key learnings from STRIVE’s baseline health facility assessments have been used to inform the implementation of the STRIVE-Tupaia platform and improve health care workers routine reporting, notification and response to vector-borne diseases in Papua New Guinea.

## Introduction

Vector borne diseases (VBD) pose an intensifying global health threat, especially in the context of resource-constrained healthcare systems, such as Papua New Guinea (PNG) [1]. Globally in 2022 there were an estimated 249 million malaria cases, an increase of 5 million cases compared with 2021[2]. PNG accounted for nearly 90% of all malaria cases and 94% of all malaria deaths in the Western Pacific Region [2], emphasising the critical need for strong surveillance systems, to monitor, track and respond to malaria in the country. Malaria control and elimination efforts remain a challenge with the emergence and spread of *Plasmodium falciparum Kelch13* C580Y mutation [3,4] as well as the first signs of *Anopheles* insecticide resistance [5]. Furthermore, arboviruses present an additional risk in PNG, with dengue viruses 1-4 currently circulating, documented outbreaks of Chikungunya [6] and widespread pyrethroid resistance in Aedes Aegypti vectors [5]. Supporting healthcare workers in VBD surveillance, notification and response is critical to develop effective strategies and implement novel interventions to curb and respond to the current malaria and arboviral trends in the country.

In PNG, healthcare services are delivered by a decentralised health system, through a combination of government and church-based health facilities as well as some private health services [7]. The main responsibility for implementing primary and secondary healthcare services in PNG lies with the subnational government, specifically the provincial- and local-level governments [8,9].The National Department of Health plays a support role in setting policies and standards and facilitating alignment in health systems and public health programs [8]. Health services in PNG are primarily funded by the national government through tax-based financing [9]. Service delivery consists of six levels as a network of national referral hospitals, provincial and district hospitals, health centres, community health posts, and aid posts [7,9]. Healthcare services provided at the community level are through the health centre and community aid posts [10]. Churches play an active role in health service delivery, especially in rural settings, however, often face resource constraints and rely on government funding [9].

Health facilities utilise the National Health Information System (NHIS) to report health data on a myriad of routine data points including malaria [11,12]. The NHIS data is comprehensive, covering all health facilities registered across PNG ([10]. Throughout 2017 and 2018, the Asian Development Bank conducted a pilot of mobile device technologies and geographic information systems in the capture and reporting of health data, initially across 184 facilities in five provinces. This pilot then expanded following an independent review to transform the NHIS, to become the electronic National Health Information System (eNHIS) in all 22 provinces. By the end of 2020, the system had been supplied to 473 facilities across 13 provinces [12]. The transition to the eNHIS aimed to increase the timeliness, completeness, quality, accessibility, flexibility, acceptability, and utility of national health data in PNG, however, challenges remain with the accessibility, uptake and use of data-for decision making [12].

The PNG National Malaria Strategic Plan (NMSP) has a focus on strengthening capacity, expanding and improving health information systems, enhancing outbreak surveillance and sentinel site surveillance. These objectives guide the generation and implementation of research ideas and activities to contribute towards evidence gaps that will strengthen the knowledge required to improve malaria and VBD control and response. Surveillance objectives are a focus of the NMSP with the current NSP 2021-2025 aiming to;

- *Strengthen capacity for epidemiological analysis for policy and decision making at all levels.*
- *Accelerate the expansion and strengthening of the eNHIS and the workforce’s capacity to utilise it*
- *Expand and strengthen outbreak surveillance and timely response for burden reduction settings*
- *Maintain sentinel site surveillance.*

The STRIVE PNG project is an implementation research program strengthening VBD surveillance and response in PNG. Beginning in 2018, the program has utilised an explicit partnership-based approach to transform existing sentinel surveillance systems to facilitate the rapid identification and containment of outbreaks, and detection of resurgent VBDs, and emerging resistance to insecticides and anti-malarial drugs [3,5]. Each sentinel site has been strategically selected based on the site’s location and capacity to provide early warning signals for rare or emerging disease threats. The sentinel surveillance system provides a value-add to the eNHIS with real-time detailed case level reporting and integrated molecular diagnostics, vector surveillance and stock management. This enables the National Malaria Control Program, to monitor trends of febrile illness, validate early warning thresholds for public health actions, and identify at risk population groups for malaria and VBDs. Data collected through the sentinel surveillance system is aggregated on a geo-spatial decision support tool called the STRIVE-Tupaia platform, that is designed to support healthcare workers in evidence-based decision making.

Our study aimed to understand the acceptability and feasibility of supporting PNGs front-line healthcare workers in VBD surveillance, adherence, reporting and notification of VBDs to inform the implementation of the STRIVE-Tupaia platform and improve the use and uptake of electronic health information systems in PNG, to support healthcare workers in evidence-based decision making towards VBD control and elimination efforts.

## Methods

### Ethics statement

STRIVE PNG received ethics approval from the PNG Institutional Review Board (1901), PNG Medical Research Advisory Committee (19.12) and Alfred Health Human Research Ethics Committee (275/19). Each of the eight study provinces provided authorisation through the governing Provincial Health Authority prior to research being conducted.

### Study sites

**Fig 1:**
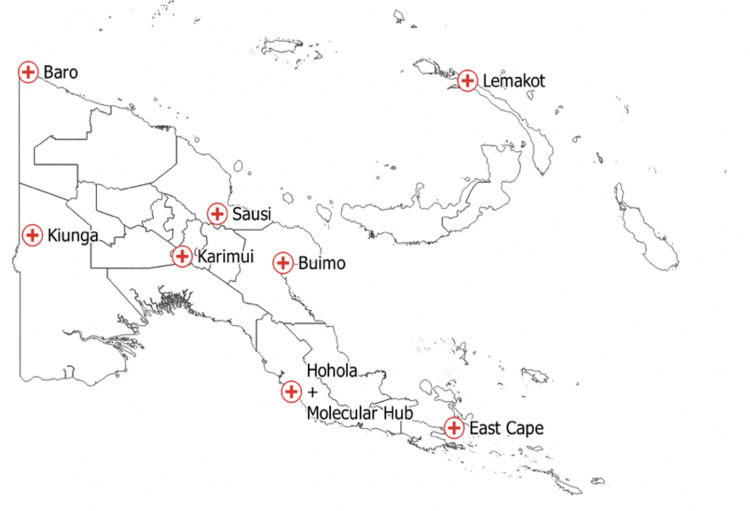
Sentinel Site Network Papua New Guinea [ 13].

PNGIMR manages the implementation of eight strategically located sentinel sites across the country in West Sepik, Morobe, National Capital District, New Ireland, Milne Bay, Madang, Chimbu and Western Province as seen in Fig 1. Since May 2018, these sites have been jointly supported by Trilateral Malaria Project and STRIVE PNG.

### Data collection

A convergent parallel mixed methods approach was undertaken through two main methodologies; semi-structured interviews (SSI) and structured observations (SO). Data collection took place at six sites over a 4-month period between 15/08/19 and 11/12/2019. Data collection was delayed at two sites due to travel restrictions associated with the SARS-CoV-2 pandemic and conducted in Western Province on 6/11/2020 and 28/07/2021 and Simbu Province on the 16/02/2021 and 18/02/2021. SSI topics were based on expected determinants of performance, VBD case management, response, reporting and notification health systems elements and co-developed with STRIVE PNG program directors and project partners. The structured observation refers to a systematic and intentional way of observing that involves using a predetermined and uniform coding scheme to identify relevant categories of observation [13]. SOs were undertaken at each sentinel site health facility and examined clinic processes, including the qualitative description of tasks involved in each of the topics outlined above, audit of equipment and consumables, standard treatment protocols, and standard operating protocols. At seven of the eight health facilities a quantification of steps, time, and patient flow of two patients presenting with a febrile illness was undertaken.

### Study population and recruitment

Purposive sampling was used to recruit participants for semi-structured interviews. Participant roles included front line health care workers providing health care services at the eight health facilities of the sentinel surveillance sites. This included clinic staff involved in the reporting and notification of disease syndromes of concern such as the sister in charge (SIC) and Officers in charge (OIC)), health managers at district and provincial levels (Provincial Malaria Control Supervisors, District Disease Control Officers), and members of the in-country clinical research team (research nurses). A courtesy letter was sent from the study teams Project Partnership Manager to each respective Provincial Health Authority (PHA) approximately a week prior to data collection occurring, informing the PHA of the planned upcoming research activities that will be occurring. Recruitment of participants then occurred during the week leading up to data collection.

### Informed consent

Written informed consent was obtained from health care providers, prior to SSIs. Interviews were conducted by trained social researchers from PNG Institute of Medical Research (ZK) and Burnet Institute (RF). Interview notes were summarised by hand, and where possible digitally recorded. The interviews were limited to a maximum of one hour and were typically undertaken following the structured observation. All names used in the manuscript are pseudonyms to protect participant confidentiality. The structured observation was conducted in collaboration with the health facility staff, typically under the guidance of the Officer in Charge (OIC). Before starting, all staff present were briefed on the research team’s objectives and activities, and verbal consent was obtained. Patients, or their guardians, also provided verbal consent for the researcher to observe the patients’ flow through the facility.

### Data analysis

Quantitative data were analysed by three researchers (YM, RF, SM) using Microsoft Excel (2019) to calculate counts and percentages. Qualitative data were thematically coded by three members of the research team (ZK, RF, YM) in NVivo12 (QSR International Pty Ltd) and analysed using categories derived from a common seven component health systems framework popularised by the WHO [14]. The data went through several rounds of coding in NVivo 12. High-level thematic categories were determined deductively through the WHO Health Systems building blocks framework and were organised in NVivo 12, laying the foundation for the coding framework. Co-analysis was then undertaken by the research team (ZK, RF, YM) to inductively identify emerging themes and sub-categories between the different building blocks to form a reflexive analysis. These included community engagement and trust, religious practises that are incorporated into daily routines and combined with health education, health facility management of staff and healthcare worker morale.

### Inclusivity in global research

Additional information regarding ethical, cultural and scientific considerations specific to inclusivity in global research is included in the Supporting Information Checklist (S1 Checklist).

## Results

We present quantitative and qualitative findings in combination to show the overall findings against each of the WHO building blocks [15]; Medicines and Technologies, Information, Financing, Service Delivery, Human Resources, and People. Additionally, the findings highlight how emerging themes influence VBD surveillance systems and offer insights into adapting interventions to be locally relevant and accepted across diverse settings. SSIs were conducted with eleven participants: (3) Nursing Officers, (6) Officers in Charge, (1) Provincial Malaria Control Supervisory, (1) Disease Control Officer. SOs were undertaken at eight sites.

### Availability and Functionality of Medicines and Diagnostic Technologies in Health Facilities – *WHO Building Block: Medicines and Technologies*

All sentinel site health facilities were accessible by road, although patients frequently accessed four of the health facilities on foot, or by boat in the case of one health facility. Information technology (IT) infrastructure at all health facilities was limited. One health facility had access to a VHF Radio and two health facilities had closed user group phones to facilitate communication between facilities and provincial health teams. None of the health facilities had internet connections established and therefore relied on health care workers personal phones and credit to communicate if VHF radios, or closed user group phones were unavailable. Table 1. Summarises the available resources across the eight sentinel site health facilities.

**Table 1:** Summary of number of sentinel site facilities with available resources.

Utility or Resource	Number of sentinel site health facilities
**Electricity**	
Functional Electricity Supply	6
Power Back up	2
Water Supply	
Piped Running Water	5
Rainwater Tanks	2
No water supply	1
**Waste Management**	
Functioning Toilets (Flushing/ Pit Latrine)	8
Incinerator	4
Open Pit	2
Other waste collection	2
**Communication**	
Internet Connection	0
VHF Radio	1
Closed User Group Phones	2
**Standard Operating Manuals**	
Standard treatment book for children	8
Standard treatment book for adults	8
Public Health Manual	2

**Fig 2:**
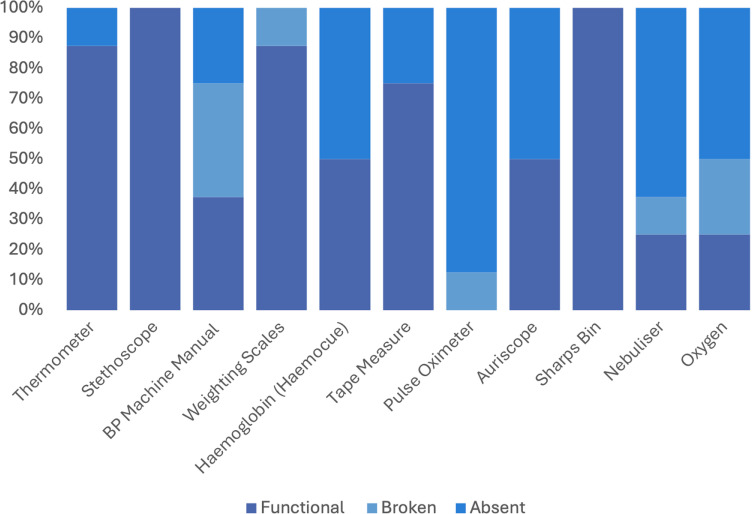
Equipment status observed at eight health facilities.

The audit of clinical equipment at each of the health facilities showed varying levels of functionality as seen in Fig 2. For example, at all health facilities functioning thermometers were present and observed however during interviews over half of the healthcare providers felt that there were an insufficient number of working thermometers available.

**Fig 3:**
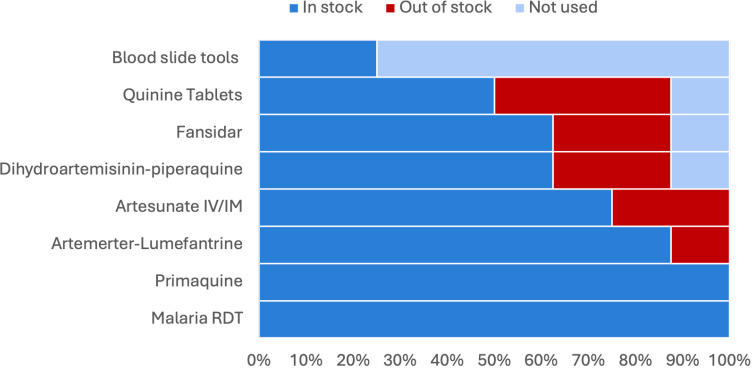
Commodities observed at eight health facilities.

Commodities required for diagnosis and treatment of malaria were available across all facilities, with seven of the eight health facilities having stock available for first line antimalarial medication and all health facilities having RDTs in stock as observable in Fig 3. Despite majority of the health facilities not having equipment and consumables required for microscopy, this was in line with the NMCP National Strategic Plan’s objective for health facilities categorised level 1-3 to have a focus on improving RDT-based diagnostic services and for health facilities level 4 and upwards to establish microscopy services for malaria diagnosis [16]. Five of the eight health facilities had Dihydroartemisinin-piperaquine in stock, the second-line treatment for uncomplicated *P. falciparum* and *P. vivax* malaria, and six facilities had Artesunate IV/ IM in stock for severe *P. falciparum* malaria. This indicates challenges with the supply chain for second line and severe malaria treatments.

*RDTs are very important and the lab test [microscopy], RDTs are done on all febrile illness patients. If no RDTs, they [clinic staff] do a blood slide - Judy, OIC, Rural Health Facility*

### Assessment of Human Resource Capacity and Staffing Levels Across Health Facilities – *WHO Building Block: Human Resources*

At the time of assessment, health workforce minimum standards were defined within the National Health Service Standards for PNG (2011-2020) [17]. Seven of the eight sentinel sites were classified as level three, and one is classified as level two. These service levels indicate the following requirements:

*Level Two* – One Community Health Worker (CHW), One Midwife and One Nursing officer covering shifts as required with shared after hours on-call arrangement. Level Two community health posts are expected to provide health services to approximately 10,000 people.
*Level Three* – Level Three comprises both Urban Clinics and Health Centres. Health Centre staff numbers and skills mix are based upon actual caseload with after hours on-call arrangements to supplement when required. Level Three health centres and urban clinics are staffed by community health workers, nursing officers and health extension officers.

**Fig 4:**
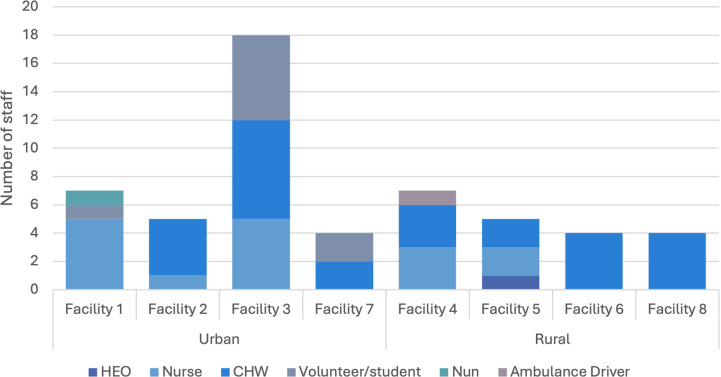
Number of staff observed at eight health facilities.

On the day of facility assessment, the total number of staff available across the eight clinics ranged from four to eighteen, with most facilities having between five and seven. The staffing breakdown at each of the facilities can be found in Fig 4. Healthcare providers noted that health facilities were not always staffed to operate at the level they had been classified, increasing the pressure on the staff to perform an array of responsibilities with limited staff available. During the observations, only one of the eight health facilities had an HEO present at the day of observation and two of the health facilities were staffed solely by CHWs. These staff shortages further reinforce the strain placed on facility operations due to limited workforce capacity to support service delivery of the catchment population.

*At the moment we are understaffed, the availability of staff at outpatient is two. If we have full staff, then we should have four staff in outpatient - Jacob, Community Health Worker, Rural Health Facility*

**Fig 5:**
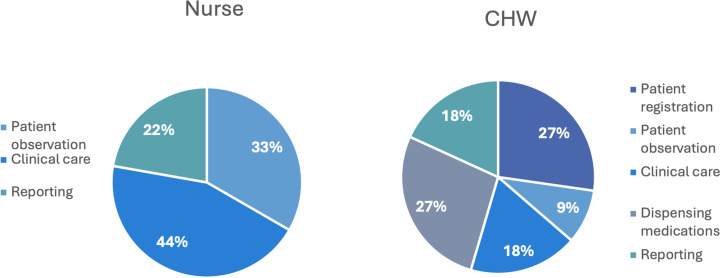
Role assignment for CHWs and nurses at the eight health facilities.

The majority of health workers observed at the facilities were either nurses or CHWs, with the main tasks associated with caring for febrile patients divided between these two cadres. Nurses were primarily focused on clinical tasks such as clinical care of patients and patient observations, whereas CHWs were more frequently responsible for patient registration, dispensing medications and reporting (Fig 5).

*My main concern is the accuracy of reporting. I cannot be here to supervise them… I have a CHW who does that [reporting] and that’s my worry, it’s supposed to be done by a nursing officer - Megan, OIC, Urban Health Facility*

Different models for training and support were implemented across the health facilities. In-house training on RDT usage and disease control was provided at one facility by the Provincial Disease Control Officer (PDCO) who trained the SICs which translated into flow-on training for nurses and CHWs. A change in the brand of malaria RDT also initiated training at another facility. Two health care workers commented on training provided by private organisations and non-governmental organisations (NGOs).

*About 3 staff here have been trained for the disease control outbreak. I came back and do in-house training to staffs on disease control, all diseases are covered in this training - Megan, OIC, Urban Health Facility*

Healthcare workers indicated several different implications of training including strengthened support to effective treatment and improvement towards patient-staff interactions. Training requirements were identified by healthcare workers for both clinical and surveillance activities. For clinical activities, a desire to have refresher training on microscopy procedures and RDT usage was highlighted.

*We need a bit of training, also on microscopy in terms of supporting RDT diagnosis as well - Jacob, CHW, Rural Health Facility*

For surveillance activities, additional training on reporting forms, outbreak response communication pathways and clarity in reporting lines was suggested.

*Ongoing refresher training on disease surveillance is important. Communication and reporting from the facility level to the provincial, and attitude change towards notifying infectious disease and be on the alert - Leila, PDCO, Provincial Health Authority*

### Challenges and variability in health information reporting systems – *WHO building block: health information systems*

At the time of the health facility assessments, seven of the health facilities were reporting monthly using the NHIS reporting forms, however the version of the reporting form differed between the health facilities with some four facilities using the updated 2019 version, two facilities using the 2018 version and one facility being unsure of the version they report on. The variation in reporting forms was also compounded by some health facilities using duplicative reporting systems (Paper-based and Electronic) which at one facility was resulting in discrepancies at the time of monthly reporting.

*We [health facility staff] try our best to be accurate. Some staff don’t report properly. Recording is done on the outpatient table however sometimes forget and then have to use memory. Sometimes registers do not align. If there is a discrepancy in the reporting, the health information officer will go through all tally sheets before signing off. - Mary, OIC, Rural Health Facility*

Several barriers to reporting were identified throughout the healthcare provider interviews. These included difficulties communicating with provincial and district healthcare officers due to limited phone credit or available phones. Across urban and rural settings, the communication systems were different, some urban settings had phones available for surveillance notification, whereas in rural settings this was uncommon.

*It would be good to have phones at all facilities to help them in reporting, if there is any need for notification. But we do have surveillance phones for notifying diseases to the PDCO- Leila, PCDO, Urban Health Facility*

Challenges with power supply and ability to charge reporting devices were identified as well as staff availability for reporting and reviewing the data collected. Four health care providers noted that quality of data entered into the monthly reporting forms could be improved, however given the high workload and competing clinical priorities for CHWs and nurses, it was noted that staff make a strong effort to report the data in a timely manner. In addition, one staff member highlighted the current gap in information reporting on VBDs emphasising that the current reporting template just includes data for malaria.

### Patient flow and clinical management of febrile illnesses – *WHO building block: service delivery*

According to patient flow observations at the seven health facilities, almost all patients presenting with a fever (11/14) had a history taken and received a malaria RDT. Of the eleven febrile patients who received a RDT, five were diagnosed with malaria and consequently treated following the PNG National Treatment Guidelines [18,19].

Findings from the interviews on clinical care were similar to structured observations of patient-flow. Eight of the eleven health providers interviewed stated they would nearly always do an RDT for a febrile patient, whereas the remaining three relied more heavily on clinical assessment. Overall, most staff interviewed had high levels of trust in the results of RDTs and felt strongly about their importance for managing febrile patients. An important change in clinical practice cited by four health providers was the use of updated guidelines for the management of malaria, leading to the appropriate use of RDTs and treatment with antimalarials.

*We use the new Malaria Protocol in 2011, it helps staff to comply with RDT as a means of testing for malaria. Only treating positive RDT with anti-malarial. Previously all febrile patients were prescribed anti-malarial, now staff and patients have [to] comply with not treating negative [cases] with anti-malarial - Leila, PDCO, Urban Health Facility*

**Fig 6:**
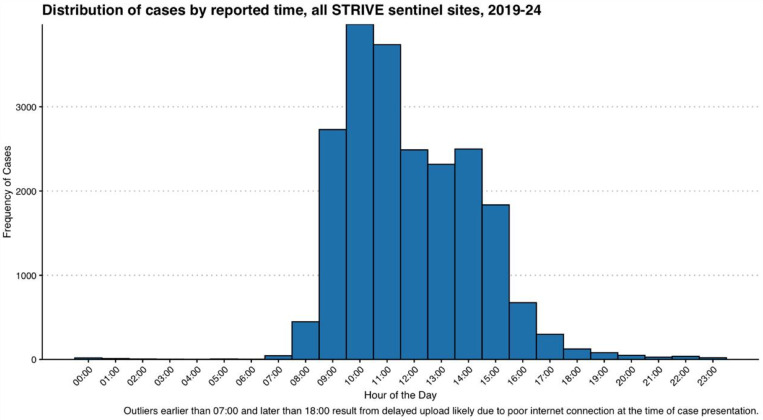
Distribution of cases by reported time across all STRIVE PNG Sentinel sites.

**Fig 7:**
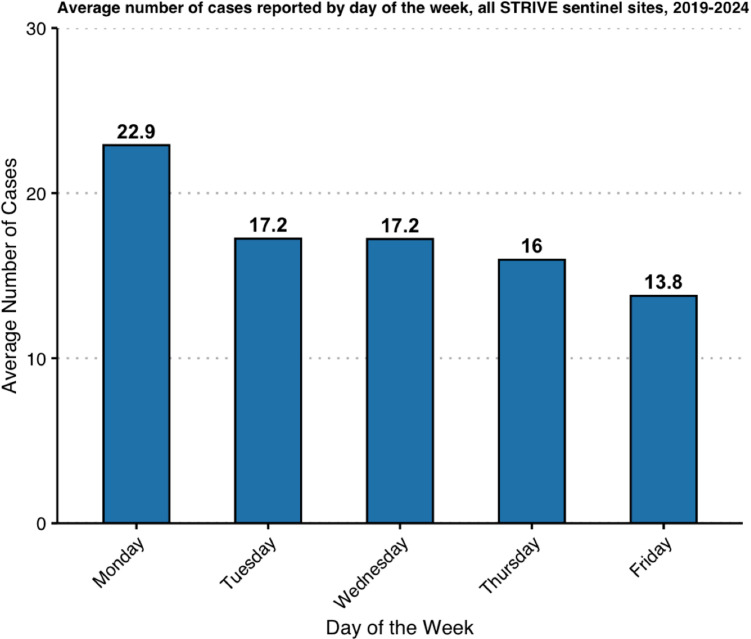
Average number of cases reported by day of the week across all STRIVE PNG Sentinel sites.

The structured observation of patient flow were undertaken first thing in the morning at all facilities, starting at 8am. As seen in Fig 6, majority of cases reported at the STRIVE PNG sentinel sites occur between 9am and 12pm each day, therefore commencing observations in the morning was important to capture patients as they arrived at the health facilities. Given the health facilities reported cases on all days, as shown in Fig 7, patient-flow observations were undertaken across a variety of weekdays (Monday-Friday).

Observation of patient flow showed considerable variation in the total time spent at the health facility for patients presenting with febrile illness, with a range of thirty one minutes to three hours and three minutes, and a median of one hour and six minutes (IQR 51 – 150) (Fig 6). Time between arrival and first clinical interaction varied from five minutes to two hours and fifty minutes (IQR 27 – 111), however clinical time was more consistent between patients, with a median of twenty three minutes and a range of nine to forty one minutes (IQR 14 – 25). Only half of patients had additional waiting times between clinical encounters, ranging from two to thirty three minutes. Urban facilities generally had longer waiting times for patients, potentially due to the higher caseload at the clinics in these settings.

**Fig 8:**
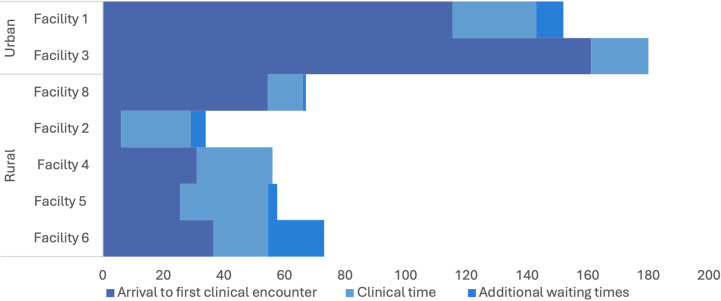
Average time spent at each health facility for 14 febrile patients.

Knowledge of standard operating procedures (SOPs) was different at each of the health facilities with varying levels of formality. The importance of SOPs was highlighted in one interview; however, it was also noted that training for use of SOPs is a critical step in order to adopt new procedures.

*There are new drugs [for malaria] in place now, patients’ response to the drugs are better with new management and protocols. However, there are no visits from doctors, we need to be upskilled and don’t want to do the wrong thing - Judy, OIC, Rural Health Facility*

In response to localised outbreaks, health care providers at each of the health facilities reported directly to PDCOs. Health care providers at one of the rural health facilities mentioned they recorded a high number of malaria cases back in 2017 and 2018. This was reported to the facility’s OIC, who then commenced testing and treatment of patients within their catchment communities and also distributed mosquito nets as a preventative measure. One health care worker explained that they receive a disease surveillance form and are required to fill this in when an outbreak occurs and then submit it to the Provincial Disease Control Office. This was not the same for other facilities. A health care provider at a separate facility mentioned that they were not given any forms to record outbreak cases and that they report directly to the PDCO. Overall, community awareness, advocacy and dissemination of health information was one of the main approaches taken by most of the health facilities when there is a disease outbreak.

*If I see an outbreak, I report to the OIC, from the OIC he reports to the district and then to the provincial level. - Miles, CHW, Rural Health Facility*

### Variability in funding sources and financial sustainability of health facilities – *WHO building block: financing*

The financing of health facilities varied among the seven interview sites. Six out of the seven health facilities received government funding sourced from district or provincial funding pools. Additionally, three facilities obtained funds from non-government sources, with two of these receiving extra funding on top of government support. One facility received funding from the private sector, while three others were supported by church funding. During interviews, some healthcare providers highlighted the challenges posed by inconsistent funding streams from district or provincial funding pools and the uncertainty surrounding the standardized allocation of funds for facility support. Two of the facilities had no user fees for clinical assessments, however the majority had outpatient fees of either one or two kina. In addition, five of the clinics had fees, ranging from two to fifty kina, attached to additional services; these included ambulance cover, ante-natal care, domestic violence, after hours, referrals, in-patient and emergency care, and a positive test result for malaria. Only one clinic had a public sign for payment requirements.

### Community engagement strategies and health education initiatives – *WHO building block: people*

Interactions between patients and health care providers, in particular CHWs and nurses, were complex across the health facilities. Since the introduction of the Malaria Treatment Protocol, one health care provider noted that staff views on treating patients had shifted and that patients are now provided with tailored health education at each consultation. Communication of health messages to patients were perceived to have improved by one health care provider, however there remained challenges with language barriers between nurses and CHWs and community members who spoke local dialects, contributing to some of the misinformation around contracting and preventing malaria.

*Staff talk to patients, if patients come with malaria they talk to them about preventative measures, talk about treatment, the communication with patient is good. Sometimes patients don’t understand the language spoken to them by the staff. Not much community involvement, not much understanding by communities in terms of infectious disease, they don’t involve themselves much*. - *Noah, Nursing Officer, Rural Health Facility*

Health awareness and education was conducted every morning as part of the ‘Tok Save’ daily routine at the facilities. Typically, this was combined with any general announcement given out by the staff to patients in the waiting area. Three of the health facilities conduct morning prayers and worship before giving health education and awareness sessions. Health education covered different diseases or if there was an outbreak identified they would notify patients during the general announcement. Interestingly, one of the health facilities had a weekly roster where each staff member gave health talks on a specific disease or health issue.

## Discussion

Our findings highlight a range of opportunities and recommendations to support front-line health workers to improve the role they play in reporting, notification and responding to vector-borne diseases in PNG.

### Options for improved reporting and notification of febrile illness

Throughout the health facility assessments, there were several local strengths and adaptations identified that could be leveraged to strengthen the surveillance, notification and reporting system for vector-borne diseases in PNG. These include:

- The presence of a Provincial Disease Control Officer (PDCO) in the province, a position that was seen to be essential for reporting and notification of febrile illness;
- Effective engagement with the local community, especially with community leaders, to strengthen community education about vector-borne diseases prevention, diagnosis and treatment;
- Research into the local burden of febrile illnesses;
- Adaptability of clinical assessments to incorporate updated guidelines.

While not always observed at the clinics during baseline assessments, these local strengths and adaptations are useful to acknowledge and build upon when planning future interventions for improving reporting and notification of febrile illness. As seen in other contexts, there is scope for narrative, story-telling forms of education based on good practice examples to help change health worker behaviours. These forms of communication can offer culturally appropriate ways of enhancing the communication of health information and improve the comprehensibility, and accessibility for healthcare workers [20].

In PNG, storytelling ‘toolkits’ are being used to harness existing knowledge and experiences across other sectors such as ‘human rights and family protection’ to guide communication and education on new laws for human rights defenders and community facilitators [21]. Insights from these sectors could be explored within the health sector to improve the applicability and learning for healthcare workers. These communication forms could significantly support the identified language barrier challenges highlighted in *People - Community engagement and Education* and be tailored for different occasions; morning ‘Tok Save’ or within patient counselling sessions.

### Training opportunities and pathways to explore

Almost all health care providers interviewed desired additional training on febrile illness. Suggested areas of focus included case management, improving patient-staff interactions, appropriate recording of febrile cases, and surveillance and notification. According to some health workers, the introduction of new guidelines and protocols was an ideal time to conduct training for facility staff.

Training and support of health care providers is an essential component of any health systems strengthening project, particularly for ensuring sustainability. As highlighted by Worsley-Tonks et al, engaging and training front-line healthcare workers to conduct surveillance could help to alleviate some of the health systems constraints present in remote and rural settings [22]. A train-the-trainer (TTT) model was one approach suggested by healthcare workers, to improve the reporting and notification of febrile illness, where key staff from each facility are trained and equipped with the knowledge and skills to train other health care providers. However, it is acknowledged that this model of training would need dedicated resources for empowering and supporting healthcare workers in ‘how to train’ others in addition to the content knowledge.

The competing responsibilities of clinical care and data reporting were also frequently mentioned. As Megan, OIC at an urban health facility, explained: *“I have a CHW who does that [reporting] and that’s my worry, it’s supposed to be done by a nursing officer.”* This reflects broader concerns about the quality of data being reported when staff are overburdened, which directly impacts continuous quality improvement (CQI) efforts and the effectiveness of vector-borne disease surveillance.

Evidence-based training packages could be developed to upskill health care providers on management of febrile patients and reporting of VBDs. This aligns with evidence generated from a systematic review of the delivery for TTT models in health and social care curricula, suggesting a blended learning approach be adopted – combining both interactive, multifaceted methods as well as learning materials [23]. These packages could be distributed through a variety of means; through TTT as above but also sent out with supplies or through supervision visits. Introducing these packages during policy rollouts or refresher training could improve CQI but also foster greater adherence to new guidelines.

There is a pressing need to identify incentives and resources that will empower clinics in running their own regular in-service training, perhaps with educational tools to support an OIC in leading staff sessions as a sustainable mechanism for staff development. It’s also important to acknowledge and plan for the foreseeable possibilities such as attrition amongst new trainers, time dedication to training and availability of resource packages to healthcare facilities [24]. As recently highlighted by Sheel and Rendell, particular consideration also needs to be given to infrastructure requirements for training and physical capacity for connectivity, with some of the study sites facing significant challenges with connectivity due to their geographical remoteness [25].

### Service delivery and standard operating procedures

In clinical management of reported fever for the fourteen febrile patients observed, there was better compliance in administering an RDT (78%) than with documenting temperature (57%). There is a high level of trust in RDTs to support clinical decisions, coupled with good understanding of new management guidelines. There is reasonable availability of the latest child health Standard Treatment Manual (STM), less so for the adult STM and least for the public health manual, with only the 2012 version seen as shown in Table 1. A range of different guidance posters were observed. Another study undertaken in PNG highlighted the information transfer from STMs to Primary Health workers. STMs were described as critical in health care workers initial training and at times when further clarity was needed around treatment regimes. Despite availability, not all health care workers used the STMs due to comprehensibility of the manuals, desire to use memory instead of manuals and difficulty in locating the answers to questions they had [26]. Concerted work on common up-to-date SOPs that are easy to interpret, and use could make a big difference, such as NDoH Public Health Manual. The version present at some of the health facilities observed was more than 10 years old, and the presence of an up-to-date guide for the public health response to disease outbreaks would be highly beneficial.

### Health information systems and outbreak response

An efficient and effective health information system is crucial for the timely reporting and notification of VBDs. While recent improvements have been made, most health care providers described challenges with reporting cases of febrile illness including technology and communication insufficiencies, competing staff priorities, and unclear reporting processes. A clear challenge to all electronic infrastructure is the lack of equitable access to internet and reception coverage across the country. Many rural areas of PNG have limited access to reception, either due to geographical barriers or restricted reception times of the day [27]. In 2019, it was noted that almost 70% of internet users resided in the cities of Port Moresby and Lae [28]. The implementation of electronic reporting systems that rely on the access to the internet need to overcome this challenge to ensure effective use of new systems, such as STRIVE-Tupaia platform, by frontline health workers. It is therefore crucial, that as the move to electronic reporting systems occurs, system are built with these challenges in mind (e.g., enabling offline data collection and storage) and that support is provided to frontline healthcare workers to establish adequate local infrastructure, processes and resources to enable the use of new digital technologies. Additionally, given the digital divide between urban and rural populations, investing in developing and implementing clear guidelines for reporting and notification of febrile illness could significantly streamline the process and save time for frontline health workers. This has been shown to be highly effective in other Western Pacific countries that have transitioned from paper based to electronic reporting for malaria surveillance [29,30], however important to also acknowledge the diversity between contextual settings and investment needed into fit-for-purpose design and implementation of new electronic platforms [25]. In addition, new technologies, such as integration of m-Supply and Tupaia, can also play an important role. Lack of staff time for reporting is a critical constraint. Any new systems need to either reduce time required and/or come with additional staff.

All staff had a good understanding of the Provincial Disease Control Officers reporting chain and several had good stories of responses. These could be a basis for locally relevant educational case studies. There was however significant variation in how staff identified, reported and responded to outbreaks. Many respondents identified non-VBD emergencies. This reinforces the need to integrate VBD surveillance and response with vaccine-preventable-diseases (e.g., pertussis, acute flaccid paralysis or measles) and others (such as respiratory syndromes typical of COVID-19 or influenza).

### Strengthening multi-disease strategies: a call for enhanced VBD management in PNG

As described by one healthcare workers *“There is a need for more training and resources for other VBDs, not just malaria”,* emphasising the current focus placed on malaria diagnosis and treatment with regards to febrile illness, at times to the detriment of other VBDs. In PNG, increased funding, programming, and activities are in place for malaria case management, surveillance, response and control in comparison to other VBDs. To improve the visibility and understanding of case management for other VBDs, strengthened guidance documents around the diagnosis, management and surveillance is needed, alongside a network of ‘champions’ to support, advocate and disseminate VBD data and awareness [25]. In addition, adequate diagnostic tools such as rapid diagnostic tests and supply chain mechanisms are required to confirm other VBDs presence and respond accordingly. The ongoing development of PNG’s National Strategic Plan for Dengue and Arboviruses will be a critical step towards achieving increased visibility and awareness for other VBDs in PNG and enable increased funding opportunities in alignment with strategic plan objectives and priorities.

## Conclusion

There is a critical need to strengthen VBD surveillance, notification and response in PNG. Our assessment across eight sentinel site health facilities in PNG highlighted opportunities to empower, strengthen and support front-line healthcare workers to improve the quality and use of data for decision making. Our results highlight the importance of applying a health systems holistic lens to system strengthening activities such as VBD surveillance, to explore innovative and cross-functional ways to adapt and embed the skills required for effective surveillance of malaria and other VBDs into routine health systems functions, workforces and communities.

## Supporting information

S1 Checklist Inclusivity in Global Research. (DOCX)

## Abbreviations

PNG: Papua New Guinea
STRIVE PNG: Stronger Surveillance and Systems Support for Rapid Identification and Containment of Resurgent or Resistant Vector Borne Pathogens in Papua New Guinea
VBD: Vector-borne Diseases
CHW: Community Health Worker
HEO: Health Extension Officer
NHIS: National Health Information System
NDoH: National Department of Health
eNHIS: Electronic National Health Information System
NGO: Non-governmental organisation
OIC: Officer in Charge
PHA: Provincial Health Authority
RDT: Rapid Diagnostic Test
SIC: Sister in Charge
SOP: Standard Operating Procedure
STM: Standard Treatment Manual
SSI: Semi-structured Interview
SO: Structured Observation
TTT: Train-the-trainer
PDCO: Provincial Disease Control Officer
VHF: Very High Frequency
CQI: Continuous Quality Improvement.
